# GR-p*K*_a_: a message-passing neural network with retention mechanism for p*K*_a_ prediction

**DOI:** 10.1093/bib/bbae408

**Published:** 2024-08-22

**Authors:** Runyu Miao, Danlin Liu, Liyun Mao, Xingyu Chen, Leihao Zhang, Zhen Yuan, Shanshan Shi, Honglin Li, Shiliang Li

**Affiliations:** Shanghai Key Laboratory of New Drug Design, School of Pharmacy, East China University of Science and Technology, No. 130, Meilong Road, Xuhui District, Shanghai, 200237, China; Innovation Center for AI and Drug Discovery, School of Pharmacy, East China Normal University, No. 3663, Zhongshan North Road, Putuo District, Shanghai, 200062, China; School of Computer Science and Technology, East China Normal University, No. 3663, Zhongshan North Road, Putuo District, Shanghai, 200062, China; Shanghai Key Laboratory of New Drug Design, School of Pharmacy, East China University of Science and Technology, No. 130, Meilong Road, Xuhui District, Shanghai, 200237, China; Shanghai Key Laboratory of New Drug Design, School of Pharmacy, East China University of Science and Technology, No. 130, Meilong Road, Xuhui District, Shanghai, 200237, China; Shanghai Key Laboratory of New Drug Design, School of Pharmacy, East China University of Science and Technology, No. 130, Meilong Road, Xuhui District, Shanghai, 200237, China; Shanghai Key Laboratory of New Drug Design, School of Pharmacy, East China University of Science and Technology, No. 130, Meilong Road, Xuhui District, Shanghai, 200237, China; Shanghai Key Laboratory of New Drug Design, School of Pharmacy, East China University of Science and Technology, No. 130, Meilong Road, Xuhui District, Shanghai, 200237, China; Shanghai Key Laboratory of New Drug Design, School of Pharmacy, East China University of Science and Technology, No. 130, Meilong Road, Xuhui District, Shanghai, 200237, China; Innovation Center for AI and Drug Discovery, School of Pharmacy, East China Normal University, No. 3663, Zhongshan North Road, Putuo District, Shanghai, 200062, China; Lingang Laboratory, No. 319, Yueyang Road, Xuhui District, Shanghai, 200031, China; Shanghai Key Laboratory of New Drug Design, School of Pharmacy, East China University of Science and Technology, No. 130, Meilong Road, Xuhui District, Shanghai, 200237, China; Innovation Center for AI and Drug Discovery, School of Pharmacy, East China Normal University, No. 3663, Zhongshan North Road, Putuo District, Shanghai, 200062, China; Department of Pain management, HuaDong Hospital affiliated to Fudan University, No. 221, West Yan'an Road, Jing'an District, Shanghai, 200040, China

**Keywords:** p*K*_a_ prediction, deep learning, retention mechanism, multi-fidelity learning

## Abstract

During the drug discovery and design process, the acid–base dissociation constant (p*K*_a_) of a molecule is critically emphasized due to its crucial role in influencing the ADMET (absorption, distribution, metabolism, excretion, and toxicity) properties and biological activity. However, the experimental determination of p*K*_a_ values is often laborious and complex. Moreover, existing prediction methods exhibit limitations in both the quantity and quality of the training data, as well as in their capacity to handle the complex structural and physicochemical properties of compounds, consequently impeding accuracy and generalization. Therefore, developing a method that can quickly and accurately predict molecular p*K*_a_ values will to some extent help the structural modification of molecules, and thus assist the development process of new drugs. In this study, we developed a cutting-edge p*K*_a_ prediction model named GR-p*K*_a_ (Graph Retention p*K*_a_), leveraging a message-passing neural network and employing a multi-fidelity learning strategy to accurately predict molecular p*K*_a_ values. The GR-p*K*_a_ model incorporates five quantum mechanical properties related to molecular thermodynamics and dynamics as key features to characterize molecules. Notably, we originally introduced the novel retention mechanism into the message-passing phase, which significantly improves the model’s ability to capture and update molecular information. Our GR-p*K*_a_ model outperforms several state-of-the-art models in predicting macro-p*K*_a_ values, achieving impressive results with a low mean absolute error of 0.490 and root mean square error of 0.588, and a high *R*^2^ of 0.937 on the SAMPL7 dataset.

## Introduction

The ionization state of drug molecules plays a pivotal role in the field of pharmaceutical research and design, with the acid–base dissociation constant (p*K*_a_) serving as a crucial parameter characterizing this state. The p*K*_a_ value reflects the tendency of a molecule to release or absorb hydrogen ions, directly correlating with its degree of ionization in physiological environments [1, 2]. The majority of pharmaceutical molecules currently on the market contain at least one acidic or basic functional group that ionizes in the human physiological environment, causing a change in charge state [3]. This alteration in ionization state further impacts the pharmacokinetics, pharmacodynamics, and toxicity of the drugs. Therefore, the development of reliable p*K*_a_ prediction tools for drug molecules is of great significance for improving the efficiency of drug discovery and design, as well as reducing the risk of drug development [4–7].

Undoubtedly, achieving accurate prediction of molecular p*K*_a_ values remains a complex problem. On the one hand, many molecules have both acidic and basic sites. Therefore, it is necessary to predict the acidic and basic p*K*_a_ values of the molecules separately. Among them, acidic p*K*_a_ refers to the process in which molecules lose hydrogen and form negative charges, while basic p*K*_a_ refers to the process in which molecules are protonated to form positive charges. On the other hand, many molecules have multiple ionization sites at the same time, which in turn generates macro-p*K*_a_ and micro-p*K*_a_. The macro-p*K*_a_ of the molecule reflects the ionization ability of the entire molecule, while the micro-p*K*_a_ of the molecule represents the protonation or de-protonation transition of specific titratable groups.

**Figure 1:**
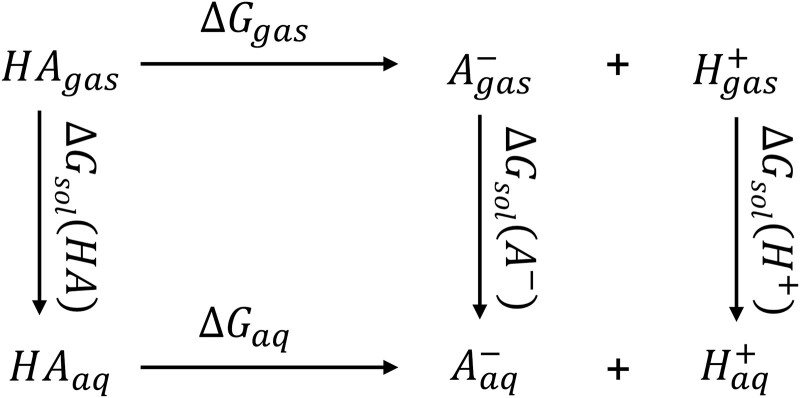
The thermodynamic cycle to calculate Δ*G*_aq_.

Notwithstanding the obstacles mentioned above in devising methodologies for molecular p*K*_a_ value prediction, a large number of methods for predicting the aqueous p*K*_a_ value of small molecules have been successfully developed, which can be divided into two classes: physical methods and knowledge-based empirical methods [8]. In physical methods, quantum mechanics (QM) approaches are employed for the calculation of molecular p*K*_a_ values, often in tandem with linear empirical correction (LEC). The incorporation of LEC is designed to mitigate systematic errors in QM predicted results, thereby contributing to improved accuracy [9]. These methods predict the p*K*_a_ value of a molecule by calculating its aqueous-phase reaction free energy at the standard state (Δ*G*_aq_). Nevertheless, given the difficulty in directly calculating the value of Δ*G*_aq_, the thermodynamic cycles are employed to address this issue, as shown in Fig. 1. By evaluating the alterations in free energy in the gas-phase and solvation processes, the standard-state free energy change within an aqueous solution could be further computed. In this class of approaches, the selection of various computational methods and basis sets exerts a profound influence on the accuracy of the calculation results. In short, although the QM method demonstrates commendable results in p*K*_a_ value calculation, its time-intensive nature and the requirement for substantial computational resources render it inadequate for the rapid and accurate prediction of p*K*_a_ values across extensive molecules datasets [10–12].

The knowledge-based empirical methods usually include two kinds of predictive models: linear free energy relationship (LFER) and quantitative structure–activity relationship (QSAR) models. The LFER approach is the oldest strategy that predicts the p*K*_a_ value of a molecule using the Hammett–Taft equation [13]. It computes the p*K*_a_ value of a molecule by leveraging the basic p*K*_a_ value of the molecular parent class and two parameters delineating substitution effects. Many commercially available software applications, such as Epik [14], use this approach to predict p*K*_a_ value which achieved relatively accurate and reliable prediction results. However, the LFER approach is limited by the available type of parent nucleus and the number of substituent empirical constants. The QSAR is a method that utilizes molecular physicochemical or structural parameters to quantitatively investigate molecular properties through mathematical and statistical approaches. Tehan *et al*. successfully constructed p*K*_a_ value predictive models for organic acids and bases, respectively, utilizing descriptors such as superdelocalizability and polarizability. However, this model is limited by the application domain, resulting in a significant decrease in performance on external test sets. [15, 16].

In recent years, the rapid development of artificial intelligence (AI) technology has greatly accelerated the process of drug discovery. The combination of QSAR and machine learning (ML) method has achieved significant success in multiple fields of drug design [17]. Baltruschat *et al*. first calculated six different features to characterize the molecule including RDKit [18] descriptors, Morgan fingerprint, the combination of them, and the *z*-transform standardized versions of these three. Subsequently, they comprehensively evaluated the six features on different models which contained random forest (RF), support vector machine (SVM), multilayer perceptron (MLP), and extreme gradient boosting (XGBoost) to compare their ability of p*K*_a_ value prediction. [4]. Yang *et al*. developed a model for predicting molecular p*K*_a_ value in different solvent environments based on an i-BonD experimental database containing 39 solvents. They constructed a structural and physical-organic-parameter-based descriptor (SPOC) which is a novel descriptor combining RDKit descriptors, molecular access system (MACCS) fingerprints [19], and ionic status labeling to represent the electronic and structural features of the molecules for p*K*_a_ value prediction. These works demonstrate that the choices of molecular descriptors and fingerprints significantly influence the accuracy of the p*K*_a_ value predicting models [1].

Simultaneously, graph-based methods have obtained great achievement in *de novo* drug design, drug–target interaction prediction, and molecular property prediction. In these methods, atoms represent nodes and chemical bonds represent edges, allowing the model to directly extract information from the molecular diagram [20–22]. Roszak *et al*. were the first to use graph convolutional networks (GCNs) to predict p*K*_a_ (DMSO) values of C–H acids in arbitrary organic molecules within milliseconds, demonstrating the potential of GCN in rapidly and accurately predicting p*K*_a_ values of molecules [23]. MolGp*K*_a_, developed by Pan *et al*. [5], leverages machine learning to discern significant chemical features and physical principles related to the acidity (or basicity) of the root atom, enabling accurate p*K*_a_ value predictions [5]. Later, Xiong *et al*. innovatively combined multi-instance learning and graph neural networks to develop Graph-p*K*_a_, a novel model offering rapid and direct microscopic p*K*_a_ value predictions based on the macroscopic p*K*_a_ value data of molecules [6]. The latest model for p*K*_a_ value prediction is the MF-SuP-p*K*_a_ jointly launched by Hou’s team. They introduced a knowledge-aware subgraph pooling strategy to improve the model’s ability to represent both local and global molecular information [7]. Overall, the graph-based neural networks have shown good performance in rapidly and accurately predicting p*K*_a_ values of molecules. Despite the demonstrated superiority of deep learning methods based on molecular graphs in predicting molecular p*K*_a_ values, existing techniques still faced limitations in their ability to handle the intricate structures and physicochemical properties of compounds, constraining the accuracy and generalization of these methods.

In the realm of molecular p*K*_a_ prediction, some methods have emerged that use descriptors derived from QM calculation. The utilization of a limited yet physically meaningful set of QM descriptors in model construction enhanced interpretability. Bannan *et al*. utilized 10 QM descriptors to construct a Gaussian process model, realizing the prediction of molecular p*K*_a_ values [24, 25]. In the preceding discussion, we explored the superiority and feasibility of Graph Neural Network methods in predicting molecular p*K*_a_ values. In addition, QM features contained information with well-defined physical–chemical significance. Hence, we posited that incorporating certain *a priori* QM features when constructing graph-based models for molecular p*K*_a_ prediction could enhance the accuracy and interpretability of predictions.

In this study, we constructed a novel p*K*_a_ prediction model named GR-p*K*_a_ (Graph Retention p*K*_a_) based on a message-passing neural network [26], which significantly enhanced the accuracy and generalization of the predicting molecular p*K*_a_ values. Considering that the predicted p*K*_a_ values for the majority of molecules in the existing database were obtained from Chemaxon, this could be a contributing factor to the suboptimal accuracy of current p*K*_a_ prediction models. To address this, we constructed a new high-quality molecular p*K*_a_ dataset and implemented a pre-training and fine-tuning strategy to enhance the alignment between the model’s predictions and experimental values [27]. The GR-p*K*_a_ approach employed five QM properties associated with thermodynamics and dynamics as molecular features to characterize molecules. In addition, the biggest highlight of this method is that the novel retention mechanism was initially integrated into the message-passing phase to enhance the model’s acquisition and updating of molecular information. The ablation study proved the effectiveness of the retention mechanism and QM features. Following a thorough evaluation on the test set and two benchmark datasets, GR-p*K*_a_ demonstrated superior performance in predicting macro-p*K*_a_ values compared to existing state-of-the-art models.

## Materials and methods

### Pre-training dataset

The pre-training dataset was assembled by leveraging data from the ChEMBL [28] and QMugs [29] datasets. Specifically, molecular p*K*_a_ values were extracted from the ChEMBL dataset, and the QM properties of molecules were acquired from the QMugs dataset. The ChEMBL database contained ~2.1 million bioactive compounds with computational p*K*_a_ values calculated using Chemaxon Marvin Suite where we could obtain a large amount of data containing molecular p*K*_a_ values. The QMugs dataset contained the biological and pharmacological properties of >665 k drug-like molecules extracted from the ChEMBL database. It also collected 42 different quantum mechanical properties calculated by both the semi-empirical method GFN2-xTB and the density-functional theory (DFT, ωB97X-D/def2-SVP). Subsequently, we matched the two databases based on ChEMBL ID to obtain a pre-training dataset comprising >410 k molecules. In the process of data partitioning, molecules were assigned to the acidic dataset when exhibiting acidic sites and to the basic dataset when possessing basic ionization sites, ensuring a distinct categorization based on molecular characteristics. This dataset was then split into an acidic dataset containing >220 k molecules and a basic dataset containing >180 k molecules. In the dataset, five quantum mechanical properties including enthalpy, free energy, highest occupied molecular orbital (HOMO) energy, lowest unoccupied molecular orbital (LUMO) energy, and HOMO–LUMO gap related to the thermodynamics and dynamics were selected for the global features of the molecule. The pre-training dataset was randomly split into the training, validation, and test sets with a ratio of 8:1:1. Due to the reality that the p*K*_a_ value of the vast majority of drug molecules is between 0 and 14, we excluded molecules with p*K*_a_ value >14 and <0 from our dataset when constructing the dataset to obtain more accurate prediction results [30].

### Fine-tuning dataset

**Figure 2:**
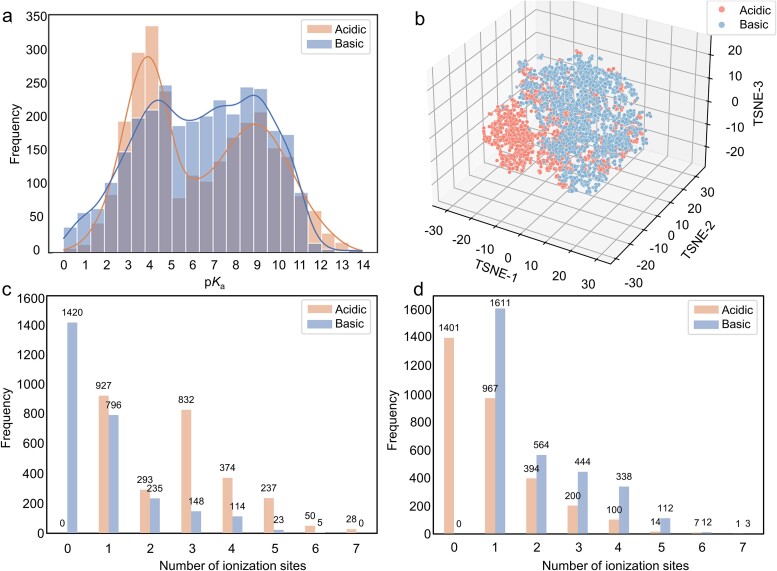
The distributions of the fine-tuning dataset. (a) The distribution of the experimental p*K*_a_ values in the acidic and basic sets, respectively. (b) The molecular chemical spatial distribution of the fine-tuning dataset. (c) The distribution of the quantity of ionization sites within the acidic dataset. (d) The distribution of the quantity of ionization sites within the basic dataset.

In pursuit of heightened predictive accuracy, we meticulously curated the fine-tuning dataset by amalgamating experimental p*K*_a_ values from diverse sources. The DataWarrior dataset, organized by Mansouri *et al*. [17], provided a high-quality and structurally diverse collection with almost 8000 molecular p*K*_a_ experimental values. In addition, we incorporated the data from Pan *et al*. [5], adding 4322 molecular experimental p*K*_a_ values to expand our dataset. Moreover, experimental p*K*_a_ values for several hundred additional molecules were obtained from pharmaceutical databases and literature [4, 31]. As many molecules were not encompassed within the QMugs dataset, the direct extraction of their five QM properties was unachievable. To address this issue, we utilized the ABT-MPNN [22] model to calculate these molecular QM properties. Finally, we segregated the dataset into distinct acidic (2740 molecules) and basic (3084 molecules) subsets to facilitate fine-tuning of the pre-trained model. In order to reduce noise in the data, each molecule only retained its most acidic and most basic p*K*_a_ values. The distribution of p*K*_a_ values and molecular chemical spatial distribution in the fine-tuning dataset are shown in Fig. 2, from where we could see that the molecules in the fine-tuning dataset occupied extensive chemical space and the p*K*_a_ values were distributed between 0 and 14. Following the SMARTS list as articulated by Pan *et al*. [5], molecules lacking identifiable ionization sites were consequently omitted from consideration. The detailed distribution depicting the quantity of ionization sites for molecules within the fine-tuning dataset is also presented in Fig. 2. It revealed that within both the acidic and basic datasets, the majority of molecules possessed a single ionizable site, and there was an overall decreasing trend in the number of molecules as the count of ionizable sites increased. We also calculated average Tanimoto coefficient based on the 166-bit MACCS fingerprints to evaluate the similarity of the molecules in the dataset. According to the low average Tanimoto coefficients of 0.1122 for acidic data and 0.0910 for basic data, the molecules in the fine-tuning dataset exhibit a substantial level of diversity. The fine-tuning dataset was randomly split into the training, validation, and test sets with a ratio of 8:1:1.

### E-p*K*_a_ dataset

**Figure 3:**
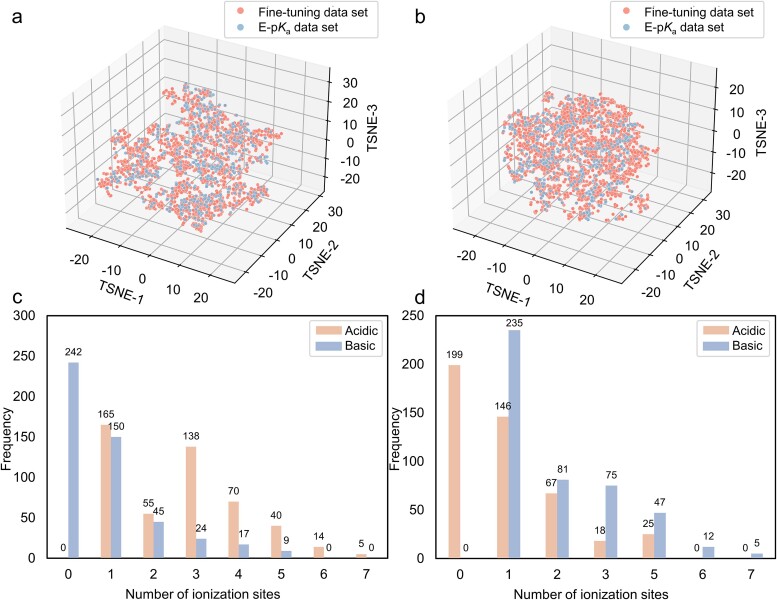
The distributions of the external E-p*K*_a_ dataset. (a) The distribution of acidic molecules in the E-p*K*_a_ dataset was compared with that of the acidic molecules in the fine-tuning dataset. (b) The distribution of basic molecules in the E-p*K*_a_ dataset was compared with that of the basic molecules in the fine-tuning dataset. (c) The distribution of the quantity of acidic molecules’ ionization sites within the E-p*K*_a_ dataset. (d) The distribution of the quantity of basic molecules’ ionization sites within the E-p*K*_a_ dataset.

To further evaluate the performance of GR-p*K*_a_, we not only evaluated it on two common datasets, SAMPL6 and SAMPL7 [8, 32], but also on our newly constructed external dataset (named E-p*K*_a_ dataset). Most of the existing p*K*_a_ value test sets focus on a certain type of molecule, such as the molecules in the SAMPL7 dataset that are all sulfonamide drug-like molecules and the molecules in the SAMPL6 dataset that are all kinase inhibitor-like compounds. In contrast, the E-p*K*_a_ dataset covers a more diverse chemical space, which includes drug-like molecules and drug molecules. We are confident that the community urgently requires a dataset of this caliber to enable a thorough comparison of various p*K*_a_ prediction methodologies. We curated E-p*K*_a_ from multiple different data sources including DrugBank [33], DataWarrior, and the literature, and removed any duplicates in the SAMPL6, SAMPL7, pre-training, or fine-tuning dataset. All molecules in the E-p*K*_a_ dataset have experimentally determined p*K*_a_ values. The E-p*K*_a_ can be further divided into an acidic dataset containing 487 molecules and a basic dataset containing 455 molecules. Figure 3 shows the chemical spatial distribution of the E-p*K*_a_ dataset and a comparison with the chemical spatial part of the fine-tuning dataset. As depicted in Fig. 3, the distribution of molecules in the E-p*K*_a_ dataset spanned extensively, covering almost the entire range of molecules in the fine-tuning dataset, both in the acidic and basic datasets. Moreover, by comparing the distribution of the number of molecular ionization sites in Figs. 2 and 3, it can be seen that the distribution trend of molecules in the E-p*K*_a_ dataset was consistent with that in the fine-tuning dataset. Therefore, we considered it appropriate to utilize the E-p*K*_a_ dataset as an external benchmark for the assessment of all methodologies.

### Retention mechanism

**Figure 4:**
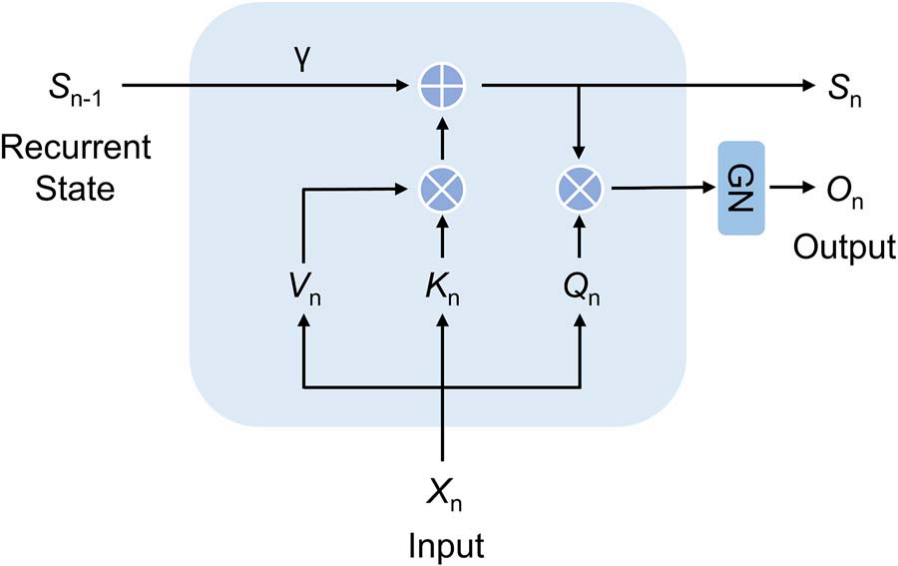
The sketch map of the retention mechanism.

Recently, Sun *et al*. proposed a novel foundation architecture for large language models named Retentive Network (RetNet), which simultaneously achieved training parallelism, low-cost inference, and good performance [34]. It replaced the standard attention mechanism with a retention mechanism based on Transformer [35]. Compared with the standard attention mechanism, RetNet introduced a position-dependent exponential decay term instead of the Softmax function to simplify the calculation process, while preserving the information from the previous step in the form of decay. The scaling invariance of GroupNorm was utilized to improve the numerical accuracy of the Retention layer. As is shown in Equations 1–2 and Fig. 4, the retention mechanism was the core of RetNet.


(1) \begin{align*} \kern1.7pc {S}_n=\gamma{S}_{n-1}+{K}_n^T{V}_n \end{align*}

(2) \begin{align*} {\displaystyle \begin{array}{c} Retention\left({X}_n\right)={Q}_n{S}_n,n=1,\cdots, \left|x\right|\end{array}} \end{align*}

Here, $n$ represents the number; ${S}_n$ represents the current state vector; ${S}_{n-1}$ represents the previous state vector; $\gamma$ is a parameter; ${X}_n$ represents the input; and ${Q}_n$, ${K}_n$, and ${V}_n$ respectively represent query matrix, key matrix, and value matrix. The hyperparameter γ serves as a key parameter within the retention mechanism, indicating the degree to which the previous state influences the current state. From Equations 1–2 and Fig. 4, we can see that the current state was only related to the previous state in the retention mechanism, which had a significant difference from the Transformer and enabled the model to focus more on local information during the learning process.

### GR-p*K*_a_ model

**Figure 5:**
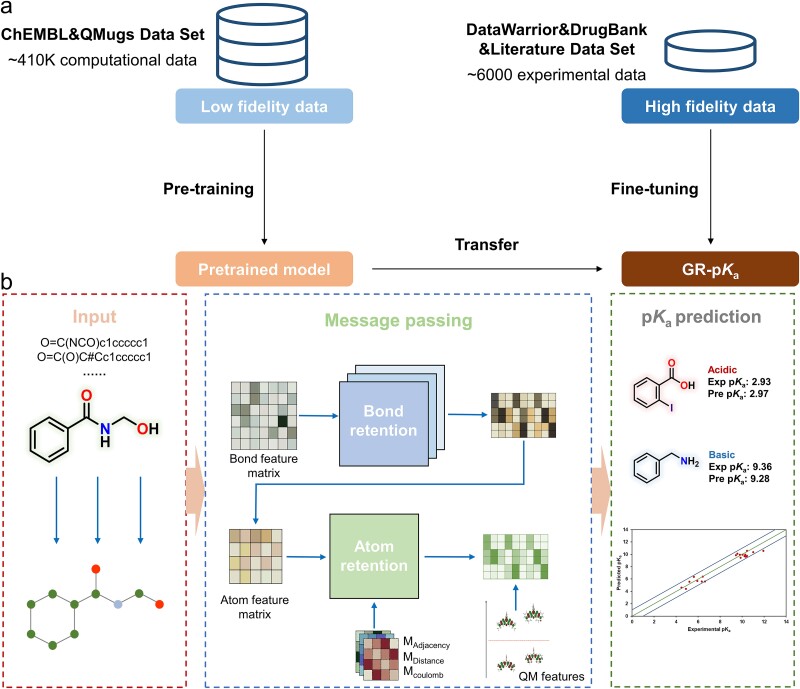
Overview of the GR-p*K*_a_ method. (a) The GR-p*K*_a_ model was built within the framework of multi-fidelity learning. (b) The prediction process consisted of three steps: molecular graph construction and featurization, message passing with the retention mechanism, and p*K*_a_ prediction.

As is shown in Fig. 5a, the architecture of the GR-p*K*_a_ model consisted of two phases: pre-training and fine-tuning. The model underwent initial training on a data-rich pre-training dataset with extensive coverage of chemical space, aiming to comprehend the QSAR between molecular structure and predicted p*K*_a_ values. Subsequently, the pre-trained model underwent additional training on the fine-tuning dataset, comprising experimental molecular p*K*_a_ values. This process facilitated the transfer of knowledge between predicted and experimental values. Overall, the distinction between the pre-training and fine-tuning processes lies in the type of p*K*_a_ values used as labels. Due to the strong correlation between calculated p*K*_a_ values and experimental p*K*_a_ values, multi-fidelity learning was implemented between them. This facilitated the expansion of the predictive range and the improvement of prediction accuracy in the model, concurrently averting the risk of negative transfer. To enhance the precision of prediction outcomes, we incorporated five well-defined QM features to characterize the compounds. Understandably, the utilization of QM features in predicting compound properties, including molecular p*K*_a_ values, was well established. Our selection of these five specific QM features was guided by two principal considerations. On the one hand, these features were intrinsically linked to the compounds’ reactivity. On the other hand, our choice was circumscribed by the constraints of the QMugs dataset, which provided restricted classes of QM properties. Simultaneously, the innovative retention mechanism was initially integrated into the message-passing phase to bolster the model’s acquisition and updating of molecular information.

As is shown in Fig. 5b, the model was predominantly composed of three components: input, message-passing neural network, and output. The input of GR-p*K*_a_ consisted of four essential elements: the node feature matrix, the bond feature matrix, the inter-atomic matrix, and five QM features. The inter-atomic matrix encompassed three parts: adjacency matrix, topological distance matrix, and Coulomb matrix. In the message-passing neural network, the bond feature matrix would first pass through the message-passing layers where the bond retention block and update functions were used to learn and update. After completing the learning of bond features through the message-passing phase, the incoming bond hidden states were summed followed by the concatenation of the atom feature matrix and a multi-head atom retention block to obtain the atomic representations. In the atom retention block, three scaled inter-atomic matrices comprising the adjacency matrix, distance matrix, and Coulomb matrix were separately attached to the head’s weight as a bias term to supply the structural and electrostatic information of the molecules. Subsequently, the acquired atomic hidden states were aggregated to produce a molecular vector, which was then integrated with the five QM features of the molecule, collectively constituting the final feature vector. At last, this consolidated feature vector was introduced to a fully connected neural network layer, dedicated to predicting the p*K*_a_ value of the molecule.

### Implementation of model and other benchmark methods

GR-p*K*_a_ utilized the PyTorch as the deep learning framework and was constructed based on the Chemprop package [36]. The MSELoss was selected as the loss function to train our model and the dropout was set to 0.1 to avoid overfitting. Furthermore, to assess the performance of GR-p*K*_a_, we conducted comparisons with traditional machine learning methods and state-of-the-art AI models. Four machine learning methods included RF, SVM, MLP, and XGBoost, where RF, SVM, and MLP were implemented through the Scikit learn package, and XGBoost was carried out by the XGBoost package. The four deep learning methods comprised Attentive FP [37], MolGp*K*_a_ [5], Graph-p*K*_a_ [6], and MF-SuP-p*K*_a_ [7]. Following the example of Baltruschat’s work, we used molecular descriptors and molecular fingerprints to characterize molecular structure, respectively. The molecular descriptors calculated by RDKit and a 4096-bit long Morgan fingerprint with a radius of 3 were computed for training the four machine learning models [4]. Attention FP is a molecular property prediction model based on attention mechanism, which is significantly recognized for its exceptional performance in property prediction. Graph-p*K*_a_, MolGp*K*_a_, and MF-SuP-p*K*_a_ are more advanced p*K*_a_ value prediction models currently. In the task of predicting molecular p*K*_a_ values, compared to other methods, these methods utilized cutting-edge molecular graph methods to accurately characterize compounds, thereby achieving accurate prediction of molecular p*K*_a_ values. In addition, the results of these methods were highly outstanding among existing methods, making them suitable benchmarks for our comparative evaluation.

### Model training and evaluation

In both the pre-training and fine-tuning processes, the dataset was all divided based on the ratio of 8:1:1. GR-p*K*_a_ was pre-trained on the computational dataset for 100 epochs with a batch size of 64 and fine-tuned on the experimental dataset for 50 epochs with the same batch size of 64. We ultimately established the optimal model based on its performance on both public datasets including SAMPL6, SAMPL7, and the E-p*K*_a_ datasets through multiple independent training sessions. As for Attentive FP and the four machine learning models, they all trained on the fine-tuning dataset. Three generic metrics for evaluating the prediction performance of the model include the coefficient of determination (*R*^2^), mean absolute error (MAE), and root mean square error (RMSE).

## Results and discussions

### Comparison with benchmark methods

**Figure 6:**
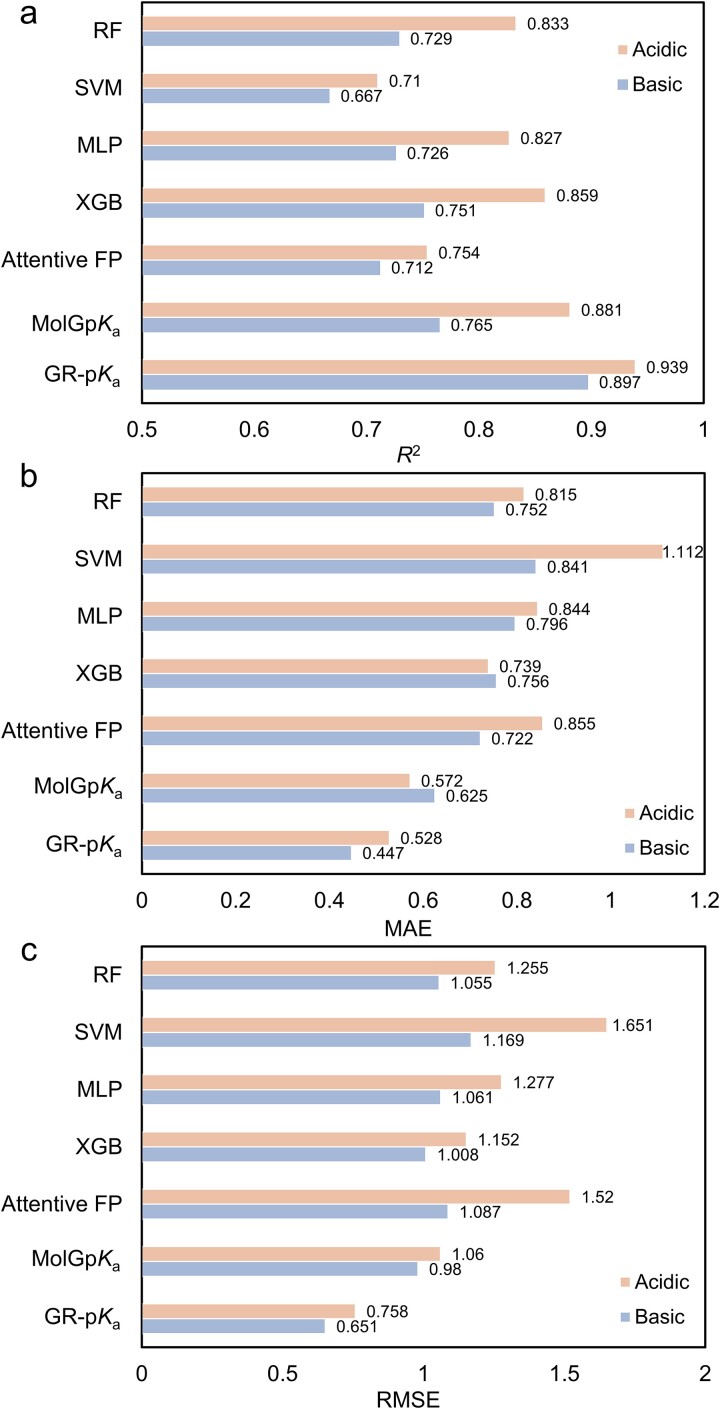
The performance of GR-p*K*_a_, four machine learning methods, and other graph-based models on the E-p*K*_a_ dataset. (a, b, c) The *R*^2^, MAE, and RMSE of those models.

To evaluate the effectiveness of GR-p*K*_a_, we implemented four traditional machine learning methods according to the methods of Baltruschat *et al*., and four advanced p*K*_a_ prediction methods as the baseline models. Six different descriptors or fingerprints containing RDKit descriptors, Morgan fingerprints, and the combination of both, as well as the scaled form of those three, were used as the indication of molecules and as the input of these machine learning methods [4]. On the fine-tuning dataset composed of molecular experimental p*K*_a_ values, the above four machine learning models were trained using a five-fold cross-validation strategy, followed by testing and comparison on the E-p*K*_a_ dataset. The performance of these four methods mainly depended on the choice of the input features including descriptors and molecular fingerprints. However, there are thousands of quantitative molecular descriptors and qualitative molecular fingerprints available to represent small molecules, the different combinations of which could result in prominent discrepancies in the same task of machine learning [38]. Since the code of MolGp*K*_a_ was not available, we were unable to retrain it using the E-p*K*_a_ dataset we built. As a result, the p*K*_a_ value prediction results were obtained by submitting the molecule in the E-p*K*_a_ dataset to the website they supplied. Similarly, the unavailable code of Graph-p*K*_a_, and the data in our test set partially overlapped with the data in their training set, so the performance of Graph-p*K*_a_ has not been evaluated and compared here. The performances of these seven models on the external dataset are shown in Fig. 6. From the three evaluation indicators, XGBoost achieved the best performance in both acidic and basic datasets in the four machine learning methods. However, even the machine learning model with the best performance in p*K*_a_ value prediction (XGBoost) still performed poorly compared to the majority of graph-based models, which further proved the superiority of graph neural networks in p*K*_a_ value prediction. The developed model GR-p*K*_a_ demonstrated the best performance among the graph-based methods, outperforming both Attentive FP and MloGp*K*_a_. Specifically, GR-p*K*_a_ got a low MAE of 0.528 and an RMSE of 0.758 with a high *R*^2^ of 0.939 on the acidic dataset and achieved a low MAE of 0.447 and an RMSE of 0.651 with a high *R*^2^ of 0.897 on the basic dataset. Nevertheless, our method performed slightly worse on the basic dataset than on the acidic dataset in terms of *R*^2^, which was also observed with all test models. The possible reasons for this phenomenon could be attributed to the slightly reduced data quality of the basic dataset in comparison to the acidic dataset. In addition, the comparatively significant deviations in the predicted results for specific molecules in the acidic dataset led to slightly higher MAE and RMSE values when compared to the basic dataset for our method. Overall, these comparison results demonstrated that our method, GR-p*K*_a_, significantly enhanced the predictive accuracy for p*K*_a_ values.

### Performance on the external datasets

Furthermore, we compared the effectiveness of different methods on the two common external datasets, SAMPL6 and SAMPL7. The SAMPL6 and SAMPL7 blind p*K*_a_ prediction challenges were respectively launched by the Drug Design Data Resource Community in 2018 and 2020. The SAMPL6 dataset consisted of 24 kinase inhibitor-like molecules with 31 experimental p*K*_a_ values and the SAMPL7 dataset contained 22 sulfonamide molecules with 20 experimental p*K*_a_ values. The SAMPL6 dataset can be further subdivided into a basic dataset containing 19 molecules and an acidic dataset containing 12 molecules, which were then predicted using the trained basic and acidic models, respectively. The molecular p*K*_a_ values contained in the SAMPL7 dataset were all acidic p*K*_a_ values of molecules.

**Figure 7:**
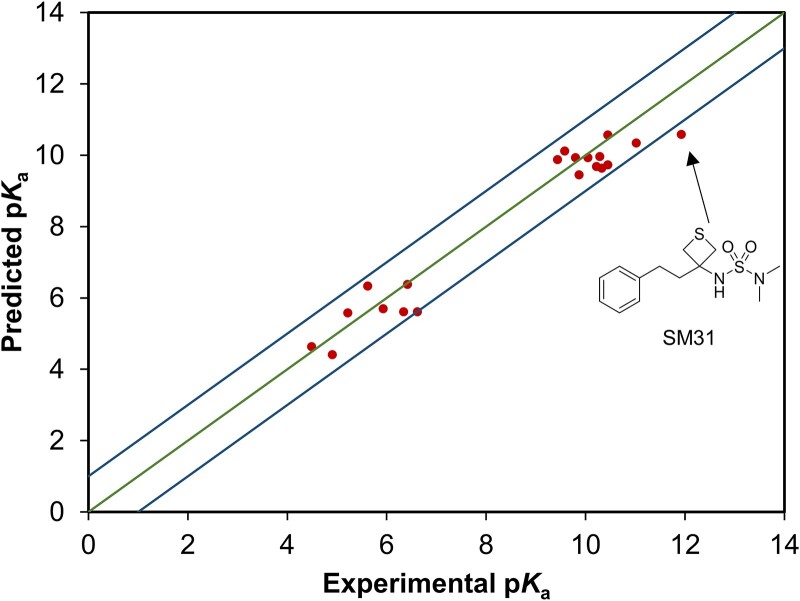
The predicted p*K*_a_ values versus experimental p*K*_a_ values on the SAMPL7 dataset (red dots). The blue lines show the errors within ±1 log unit. The green line represents that the predicted values are equal to the experimental values. The SM31 has the largest absolute error around the SAMPL7 dataset.

The performances of those models on the SAMPL6 and SAMPL7 datasets are shown in Table 1. From Table 1, it can be seen that GR-p*K*_a_ exhibited comparable performance on the SAMPL6 and the best performance on the SAMPL7 dataset compared to all other models. Our model achieved a low MAE of 0.490 and an RMSE of 0.588, along with a high *R*^2^ of 0.937 on the SAMPL7 dataset. These results underscore the advantages of GR-p*K*_a_ over existing models. As shown in Fig. 7, with the exception of the largest absolute error for SM31 (1.35), the discrepancies between the predicted and experimental values for all other molecules fell within ±1 log unit. This performance was in line with the lower RMSE and higher *R*^2^ value obtained by our model. In addition, the MF-SuP- p*K*_a_ method, which also utilized the pre-training and fine-tuning strategies, ranked second in the performance on the SAMPL7 dataset. This indicated that the use of pre-training and fine-tuning strategies provided substantial benefits for the prediction of p*K*_a_ values. Furthermore, the four machine learning methods were also evaluated on these two datasets; however, their performance was notably inferior to that of the graph-based methods (Supplementary Table S1 available online at http://bib.oxfordjournals.org/).

**Table 1:** The performance of GR-p*K*_a_ and other graph-based models on the SAMPL6 and SAMPL7 datasets

Dataset	Model	*R* ^2^	MAE	RMSE
SAMPL6	Attentive FP	0.588	1.243	1.727
	MolGp*K*_a_^(a)^	0.904	0.567	0.835
	Graph-p*K*_a_^(b)^	0.899	0.670	0.856
	GR-p*K*_a_	0.905	0.664	0.834
SAMPL7	Attentive FP	0.668	1.101	1.345
	MolGp*K*_a_^(a)^	0.824	0.804	0.986
	Graph-p*K*_a_^(b)^	0.867	0.659	0.854
	MF-SuP-p*K*_a_^(c)^	0.879	0.656	0.816
	GR-p*K*_a_	0.937	0.490	0.588

The model which has the best performance and its “MAE,” “RMSE,” and “*R*^2^” values have been highlighted in bold

^a^The result was obtained on the web server of MolGp*K*_a_ (http://xundrug.cn/molgpka)

^b^The result was obtained on the web server of Graph-p*K*_a_ (https://pka.simm.ac.cn/en/)

^c^The predicted values are provided in the original article of MF-SuP-p*K*_a_

### Ablation study

In this section, we evaluated the contribution of each component to the model performance through the ablation study. Three variants of GR-p*K*_a_ were defined, with the first variant removing multi-fidelity learning (MFL), QM features, and retention mechanism (denoted as N), the second variant removing the QM features and retention mechanism (denoted as Q), and the third variant only removing the retention mechanism (denoted as R). In all variants, we used attention mechanism as a replacement for retention mechanism. As is shown in Table 2, we tested the effectiveness of all variant strategies on acidic and basic data from the E-p*K*_a_ dataset, respectively. By comparing models N and Q, we can see that the introduction of MFL significantly improved the model’s performance, especially on basic datasets, with an increase of up to 27.14% (RMSE). This indicated that the strategy of transferring predicted values to experimental values is effective for predicting molecular p*K*_a_ values. The comparison between models Q and R revealed that introducing QM features also led to a slight improvement in the model’s performance, aligning with previous studies that utilized QM descriptors for predicting molecular p*K*_a_ values [24, 25]. As a novel strategy, the effectiveness of the retention mechanism was further validated through comparisons between models R and GR-p*K*_a_. Overall, the synergistic application of three distinct strategies led to an enhancement of 29.22% in model performance on the acidic dataset and a substantial improvement of 38.87% on the basic dataset.

**Table 2:** The ablation results on the external E-p*K*_a_ dataset

Task	Variant	Strategy			Metric			Relative gain (RMSE)
		MFL	QM	Ret	*R* ^2^	MAE	RMSE	
Acidic	N	×	×	×	0.878	0.764	1.071	–
	Q	√	×	×	0.906	0.686	0.936	12.61%
	R	√	√	×	0.920	0.677	0.866	19.14%
	GR-p*K*_a_	√	√	√	0.939	0.528	0.758	29.22%
Basic	N	×	×	×	0.722	0.720	1.065	–
	Q	√	×	×	0.854	0.596	0.776	27.14%
	R	√	√	×	0.871	0.594	0.726	31.83%
	GR-p*K*_a_	√	√	√	0.897	0.447	0.651	38.87%

Three strategies used in GR-p*K*_a_ model. MFL, multi-fidelity learning; QM, quantum mechanical features; Ret, retention mechanism.

Based on the above analysis, all three modules were effective in improving the model’s performance. The incorporation of QM features, with clear physical and chemical meanings, proved helpful for the accurate prediction of molecular p*K*_a_ values. Moreover, the integration of multi-fidelity learning expanded the model’s predictive capabilities, smoothing the transition from predicted to experimental values. It is widely recognized that the fewer chemical bonds between two atoms, the stronger the interactions between them, highlighting the importance of focusing on the local information within molecules. In the retention mechanism, the current state is solely dependent on its previous state. As the feature updating process advances, the impact of early states on the current state gradually decreases. The retention mechanism aligned with this fundamental chemical principle to a certain degree, leading us to conclude that it offers advantages over the attention mechanism in capturing molecular information. The ablation study’s outcome substantiated our hypothesis, demonstrating that the inclusion of the retention mechanism during the message-passing phase significantly improved the model’s ability to update and learn chemical information. This finding also implied that the retention mechanism holds promise for enhanced utility in the field of molecular property prediction.

## Conclusion

In this work, we developed a molecular p*K*_a_ value predictor using a message-passing neural network named GR-p*K*_a_. Compared to the traditional QSAR models reliant on manually encoded molecular descriptors or fingerprints, our approach leveraged message-passing layers to aggregate the information from neighborhoods and update the hidden states of the target node. To address the intrinsic challenge of limited experimental p*K*_a_ data, we utilized the multi-fidelity learning strategy to improve the generalization and accuracy of the model, successfully achieving the transfer from calculated values to experimental values. We systematically evaluated the performance of the model on external datasets and two benchmark datasets, and the results demonstrated that GR-p*K*_a_ exhibited outstanding performance on both datasets. The retention mechanism was first brought into the field of molecular property prediction and achieved good performance. The ablation experiment demonstrated the effectiveness of introducing the novel retention mechanism and QM features with clear physicochemical meanings to the model. We hope the success of these strategies will also inspire researchers and provide a reliable solution for them when encountering similar problems.

While our method exhibited advantages over existing approaches, limitations persisted due to data scarcity, impeding the assembly, construction, and refinement of microscopic p*K*_a_ datasets. Although proficient in predicting macroscopic acidic and basic p*K*_a_ values, our method falls short in forecasting microscopic counterparts. The accurate prediction of microscopic p*K*_a_ values is essential for structural modifications, but such prediction remains elusive. Future model development should focus on predicting both macroscopic and microscopic p*K*_a_ values, depending on the availability of additional experimental data.

**Key Points**

- The GR-p*K*_a_ is a cutting-edge p*K*_a_ prediction model based on message-passing neural network, which enables the effective integration and processing of molecular information to enhanced p*K*_a_ value predictions.
- A multi-fidelity learning strategy is introduced to effectively integrate quantum mechanical properties with empirical data, improving the accuracy and robustness of molecular p*K*_a_ predictions, especially in the context of limited experimental data.
- The GR-p*K*_a_ model uniquely integrates five quantum mechanical properties related to molecular thermodynamics and dynamics as molecular features, providing a deeper and more scientifically rigorous characterization of the molecules.
- A pioneering retention mechanism is introduced within the message-passing phase, enhancing accuracy by focusing on critical molecular interactions during the learning process.

## Supplementary Material

V2_pka_SI_bbae408

## Data Availability

The source code of GR-p*K*_a_ and the weights for the best-trained model can be found at https://github.com/yzjyg215/GR-pKa.
