# Supplementary material for: GR-pKa: a message-passing neural network with retention mechanism for pKa prediction
Source: Brief Bioinform. 2024 Aug 22;25(5):bbae408. doi: 10.1093/bib/bbae408 (PMC11339865; doi:10.1093/bib/bbae408)
Supplement: V2_pka_SI_bbae408 [file v2_pka_si_bbae408.docx]

**Supporting Information for**

**GR-p*K*_a_: A message-passing neural network with retention mechanism for p*K*_a_ prediction**

Runyu Miao *et al.*

* Corresponding author. Email: hlli@ecust.edu.cn, slli@hsc.ecnu.edu.cn

This word file includes:

Table S1

Table. S1. The performance of four machine learning methods on the SAMPL6 dataset and the SAMPL7 dataset

| **Dataset** | **Model** | ***R*^2^** | **MAE** | **RMSE** |
| --- | --- | --- | --- | --- |
| **SAMPL6** | RF | 0.290 | 1.791 | 1.979 |
|  | SVM | -0.166 | 2.270 | 2.536 |
|  | MLP | -0.108 | 2.308 | 2.465 |
|  | XGB | 0.180 | 1.947 | 2.213 |
| **SAMPL7** | RF | -0.348 | 1.516 | 1.882 |
|  | SVM | -0.671 | 1.658 | 2.125 |
|  | MLP | -0.027 | 1.413 | 1.643 |
|  | XGB | -0.553 | 1.760 | 2.075 |
